# Noradrenergic modulation of saccades in Parkinson’s disease

**DOI:** 10.1093/braincomms/fcae297

**Published:** 2024-09-01

**Authors:** Isabella F Orlando, Frank H Hezemans, Rong Ye, Alexander G Murley, Negin Holland, Ralf Regenthal, Roger A Barker, Caroline H Williams-Gray, Luca Passamonti, Trevor W Robbins, James B Rowe, Claire O’Callaghan

**Affiliations:** Brain and Mind Centre and School of Medical Sciences, Faculty of Medicine and Health, University of Sydney, Sydney 2050, Australia; MRC Cognition and Brain Sciences Unit, University of Cambridge, Cambridge CB2 7EF, UK; Department of Clinical Neurosciences and Cambridge University Hospitals NHS Trust, University of Cambridge, Cambridge CB2 0SZ, UK; Department of Clinical Neurosciences and Cambridge University Hospitals NHS Trust, University of Cambridge, Cambridge CB2 0SZ, UK; Department of Clinical Neurosciences and Cambridge University Hospitals NHS Trust, University of Cambridge, Cambridge CB2 0SZ, UK; Department of Clinical Neurosciences and Cambridge University Hospitals NHS Trust, University of Cambridge, Cambridge CB2 0SZ, UK; Division of Clinical Pharmacology, Rudolf-Boehm-Institute for Pharmacology and Toxicology, University of Leipzig, Leipzig 69978, Germany; John van Geest Centre for Brain Repair, Department of Clinical Neurosciences, University of Cambridge, Cambridge CB2 0SZ, UK; Wellcome Trust—Medical Research Council Stem Cell Institute, University of Cambridge, Cambridge CB2 0AW, UK; John van Geest Centre for Brain Repair, Department of Clinical Neurosciences, University of Cambridge, Cambridge CB2 0SZ, UK; Department of Clinical Neurosciences and Cambridge University Hospitals NHS Trust, University of Cambridge, Cambridge CB2 0SZ, UK; Department of Psychology, University of Cambridge CB2 3EA, Cambridge UK; Behavioural and Clinical Neuroscience Institute, University of Cambridge, Cambridge CB2 3EA, UK; MRC Cognition and Brain Sciences Unit, University of Cambridge, Cambridge CB2 7EF, UK; Department of Clinical Neurosciences and Cambridge University Hospitals NHS Trust, University of Cambridge, Cambridge CB2 0SZ, UK; Brain and Mind Centre and School of Medical Sciences, Faculty of Medicine and Health, University of Sydney, Sydney 2050, Australia

**Keywords:** noradrenaline, atomoxetine, saccades, Parkinson’s disease, locus coeruleus

## Abstract

Noradrenaline is a powerful modulator of cognitive processes, including action decisions underlying saccadic control. Changes in saccadic eye movements are common across neurodegenerative diseases of ageing, including Parkinson’s disease. With growing interest in noradrenergic treatment potential for non-motor symptoms in Parkinson’s disease, the temporal precision of oculomotor function is advantageous to assess the effects of this modulation. Here, we studied the effect of 40 mg atomoxetine, a noradrenaline reuptake inhibitor, in 19 people with idiopathic Parkinson’s disease using a single dose, randomized double-blind, crossover, placebo-controlled design. Twenty-five healthy adult participants completed the assessments to provide normative data. Participants performed prosaccade and antisaccade tasks. The latency, velocity and accuracy of saccades, and resting pupil diameter, were measured. Increased pupil diameter on the drug confirmed its expected effect on the locus coeruleus ascending arousal system. Atomoxetine altered key aspects of saccade performance: prosaccade latencies were faster and the saccadic main sequence was normalized. These changes were accompanied by increased antisaccade error rates on the drug. Together, these findings suggest a shift in the speed-accuracy trade-off for visuomotor decisions in response to noradrenergic treatment. Our results provide new evidence to substantiate a role for noradrenergic modulation of saccades, and based on known circuitry, we advance the hypothesis that this reflects modulation at the level of the locus coeruleus–superior colliculus pathway. Given the potential for noradrenergic treatment of non-motor symptoms of Parkinson’s disease and related conditions, the oculomotor system can support the assessment of cognitive effects without limb-motor confounds on task performance.

## Teaser

Orlando *et al*. show that atomoxetine (a noradrenaline reuptake inhibitor) modulates saccade behaviour in Parkinson’s disease. This is significant considering the growing interest in noradrenergic treatment for non-motor symptoms in Parkinson’s and related disorders, providing the first evidence of a noradrenergic contribution to eye movement decision processes in Parkinson’s disease.

## Graphical Abstract

**Graphical Abstract:**
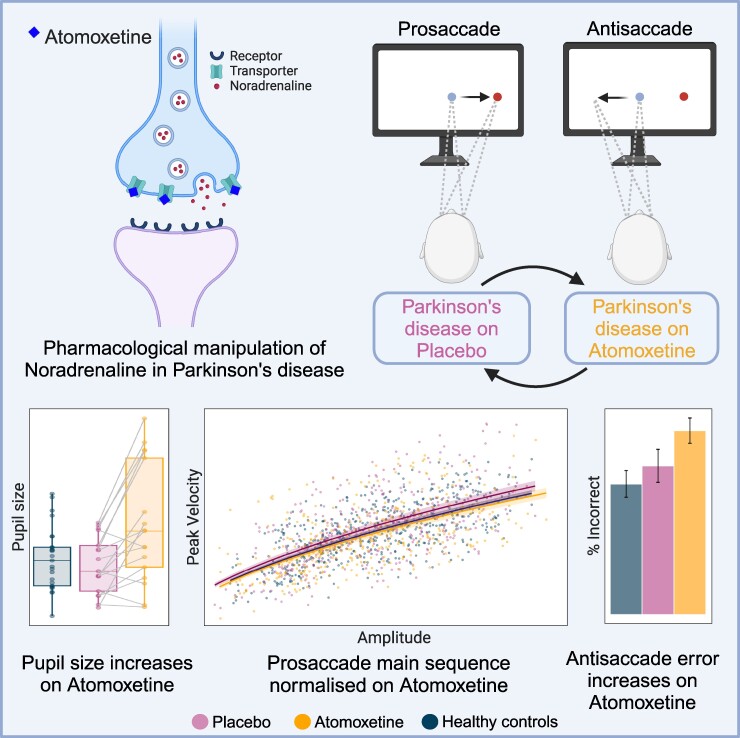

See A. Antoniades (https://doi.org/10.1093/braincomms/fcae337) for a scientific commentary on this article.

## Introduction

The oculomotor system sits at the interface of a perception-action cycle that integrates visual information from the environment with an organism’s current internal state, facilitating the decisions underlying adaptive behaviour. Saccadic eye movements provide a means of exploring the visual world, subject to reflexive and higher-order cognitive processes. Control of saccades involves the visual, prefrontal and parietal cortices, basal ganglia, thalamus, cerebellum, brainstem reticular formation and superior colliculus.^1^ Distributed neural processes across this network enable interactions between an organism’s environment, goals and behaviour.^2,3^ The oculomotor output of the superior colliculus integrates this exogenous and endogenous information, enabling optimal decisions about where and when to direct a saccade.^4^ In this way, eye movements reflect the dynamics of decision processes, including information accumulation, evaluation, deliberation and choice.^5^

Eye movement abnormalities are not a defining feature of Parkinson’s disease, but they are nonetheless commonly affected, with marked individual differences.^6-10^ With progression of motor and cognitive impairment, prolonged saccadic latencies, fragmented, hypometric saccades and increased size and frequency of square wave jerks may be observed.^10-14^ However, the latency and accuracy of saccades have been of particular interest in identifying the effect of disease and treatment on decision-making processes. For example, on more cognitively demanding tasks, including memory-guided saccades and antisaccades, inhibitory deficits are evident early in the illness and worsen with disease progression and cognitive impairment.^13,15-18^

Saccade deficits in Parkinson’s disease are often interpreted in light of dopamine cell loss in the substantia nigra pars compacta. In Parkinson’s disease, reduced substantia nigra pars compacta dopaminergic input to the caudate results in a loss of inhibitory tone from the globus pallidus externus; this permits excessive outflow from the subthalamic nucleus to the substantia nigra pars reticulata, leading to tonic inhibition over the superior colliculus which controls the downstream premotor circuit for saccade initiation.^9,19^ However, the diversity of saccade deficits and their heterogeneity among individuals with Parkinson’s disease suggests mechanisms extrinsic to the basal ganglia and beyond dopaminergic modulation. Noradrenergic contributions to the phenotype of Parkinson’s disease are of particular interest, because of therapeutic opportunities.^20^

Noradrenergic projections from the locus coeruleus innervate intermediate layers of the superior colliculus.^21-23^ In the non-human primate visual system, noradrenergic fibres target regions involved in spatial processing and visuomotor responses (i.e. the tecto-pulvinar-extrastriate visual structures).^23-25^ Other regions of the oculomotor network, including the thalamus, prefrontal, parietal and visual cortices, receive dense noradrenergic innervation from the locus coeruleus.^26^ Noradrenaline supresses spontaneous firing and enhances stimulus-evoked firing, producing a net increase in signal-to-noise ratio.^27,28^ In sensory circuits, a ‘gating’ effect is observed, where noradrenaline induces a robust cellular response to otherwise subthreshold visual stimuli.^29,30^ Together, noradrenaline modulates visual-spatial attention by enhancing perceptual sensitivity.^31^

The locus coeruleus is one of the earliest sites of pathology in Parkinson’s disease,^32^ with severe deficits in the symptomatic stages.^33^ This noradrenergic deficit is implicated in diverse cognitive and neuropsychiatric symptoms.^20,33-38^ In light of this, there is growing interest in noradrenergic treatment for non-motor symptoms in Parkinson’s disease and related disorders.^39,40^ Oculomotor measures are particularly relevant in Parkinson’s disease, as they offer a means of assessing decision mechanisms and treatment effects without the confounding effect of limb akinetic-rigidity. Here, we test the hypothesis that atomoxetine, a noradrenaline reuptake inhibitor, modifies oculomotor control in Parkinson’s disease. We measured prosaccade and antisaccade task performance in people with Parkinson’s disease using a single dose double-blind, crossover, placebo-controlled design.

## Materials and methods

This study was part of a broader project: study design and participant characteristics overlap with previous publications.^37,38,41^ The study was approved by the local ethics committee (REC 10/H0308/34), and all participants provided written informed consent according to the Declaration of Helsinki. The study was retrospectively registered on ISRCTN (ISRCTN46299660).

### Participants

Nineteen people with Parkinson’s disease were recruited through the University of Cambridge Parkinson’s disease research clinic and Parkinson’s UK volunteer panels. All participants met UK Parkinson’s Disease Society Brain Bank diagnostic criteria, were aged between 50 and 80 years and had no contraindications to atomoxetine or 7 T MRI. No participant met criteria for dementia.^42^ Twenty-six age-, sex- and education-matched healthy control participants were recruited. Control participants were screened for a history of psychiatric or neurological disorders and were not taking psychoactive medications. One control participant was excluded from all analyses due to inadequate eye tracking data.

### Study procedure

Participants with Parkinson’s disease were tested over three sessions. In the first session, they completed MRI scanning and clinical assessment of cognition and motor function (see Table 1 and Supplementary Table 1). The second and third sessions comprised a double-blind, placebo-controlled crossover design, where participants were block randomized to receive 40 mg of atomoxetine or placebo. Session two and three visits were ≥6 days apart [mean = 7.4 days; standard deviation (SD) 1.8; range 6–14]. Blood samples were taken 2 h post-drug/placebo administration, corresponding to the predicted peak in plasma concentration.^43^ Mean plasma concentration^44^ was 264.07 ng/mL after atomoxetine (SD = 124.50 ng/mL, range: 90.92–595.11 ng/mL) and 0 ng/mL after placebo. Participants then commenced an experimental task battery including the oculomotor tasks reported here. Blood pressure and pulse rate were taken across the sessions, and visual analogue scales were administered to monitor mood/arousal levels (see Supplementary material, Supplementary Table 2 and Supplementary Figs 1 and 2). All sessions were completed at a similar time of day, with participants on their regular anti-parkinsonian medications. Control participants provided normative data, and they were tested in a single session and did not undergo the drug manipulation.

**Table 1:** Data are presented as mean (SD)

	Parkinson’s disease	Controls	BF	*P*
Age (years)	67.11 (7.05)	65.40 (5.42)	0.42	0.388
Education (years)	14.05 (2.27)	14.64 (3.16)	0.36	0.477
Male/female	15/4	14/11	1.15	0.204
MMSE	29.47 (0.70)	29.80 (0.50)	1.10	0.093
MoCA	28.11 (1.76)	28.52 (1.39)	0.41	0.403
ACE-R				
Total score	94.89 (3.71)	97.56 (3.23)	**3**.**65**	0.**017**
Attention and orientation	17.84 (0.37)	17.96 (0.20)	0.62	0.225
Memory	23.68 (1.97)	25.04 (1.18)	**7**.**65**	0.**012**
Fluency	12.00 (2.08)	12.76 (1.61)	0.63	0.196
Language	25.84 (0.50)	25.88 (0.44)	0.31	0.795
Visuo-spatial	15.63 (0.50)	15.80 (0.65)	0.43	0.333
MDS-UPDRS				
I: non-motor experiences	9.00 (4.18)			
II: motor experiences	12.63 (4.26)			
III: motor examination	28.42 (11.60)			
IV: motor complications	0.47 (0.96)			
Total score	50.58 (17.20)			
Hoehn and Yahr stage	2.26 (0.45)			
Disease duration (years)	4.15 (1.72)			
Levodopa equivalent daily dose (mg/day)	644.55 (492.81)			

Comparisons of patient and control groups were performed with independent samples *t*-tests or contingency tables as appropriate. BF > 3 and *P* < 0.05 are bolded and considered significant.

MMSE, Mini-Mental State Examination; MoCA, Montreal cognitive assessment; ACE-R, revised Addenbrooke’s Cognitive Examination; MDS-UPDRS, Movement Disorders Society Unified Parkinson’s Disease Rating Scale.

### Oculomotor assessments

Pupil size and eye movements were recorded using an EyeLink 1000 portable duo eye tracker at a sampling rate of 500 Hz (see Supplementary material for full set-up details). The tasks were programmed using EyeLink Experiment Builder software, with further pre-processing conducted in R (version 4.2.1, R Core Team, 2022^45^). Our analyses used saccade detection, velocity and amplitude calculations from the standard EyeLink algorithms.

#### Resting pupil diameter

Pupil diameter was recorded over a 3 min period at rest. Participants were asked to maintain fixation on a central crosshair. We first assessed signal quality by dividing each participant’s samples into 15 s time windows and removing windows with >15% of samples missing. Standard pre-processing followed: we removed 100 ms before and after blinks^46^ and linearly interpolated the missing traces and then applied Hanning window smoothing.^47^ Pupil diameter was calculated in arbitrary EyeLink units and *Z*-scored. We removed extreme samples that were ±3 SD from the mean and averaged across all surviving time windows.

#### Prosaccade and antisaccade tasks

For the prosaccade and antisaccade tasks, participants were presented with a red circle (the target) on a black background. Each trial began with the target presented centrally for a duration varying between 800 and 1200 ms. The target stimulus then disappeared, and following a 200 ms gap, it appeared on the left- or right-hand side of the screen. The target had six possible positions on the horizontal axis, presented in a randomized order. From the centre (0°), target locations were ±3°, ±5°, and ±7°. Both tasks included 24 trials, with equal amounts of each eccentricity. In the prosaccade task, participants were instructed to direct their gaze towards the target. In the antisaccade task, participants were instructed to direct their gaze away from the target to the other (blank) side of the screen.

For analysis, in each trial, we identified the primary saccade—i.e. the first valid saccade following the target’s appearance on either side of the screen.^48,49^ The primary saccade was defined by an amplitude between >1.5° and <10° (i.e. to rule out microsaccades and saccades too extreme to remain on the screen^50,51^) and a latency between > 90 and <2500 ms (i.e. to rule out anticipatory saccades^52,53^ and inattention^54^).

Prosaccade analysis was conducted on the first valid saccade directed towards the target. Antisaccade analysis was performed on the first valid saccade regardless of direction and identified as a correct antisaccade if the direction was opposite to the target. Prosaccade trials with latencies, peak velocities, amplitudes or peak velocity residuals more extreme than ±2.5 SD from the participant’s mean were removed; similarly, this was done for antisaccade trials with respect to latency.

For prosaccades, we measured velocity and amplitude throughout the saccade, by normalizing the duration of each saccade and then dividing into 10 time bins. For each prosaccade, we measured response latency (i.e. time from target display to initiation of the primary saccade), peak velocity (i.e. maximum velocity during the saccade) and absolute amplitude (i.e. angular distance travelled during the saccade). There is a well-established relationship known as the ‘main sequence’ where a saccade’s velocity increases as amplitude increases.^55^ To account for this, we used the peak velocity residuals as our velocity measure of interest, calculated by regressing amplitude scores against velocity scores.^56^ For the antisaccade task, we calculated error rates as the proportion of incorrectly directed antisaccades from the total completed trials (i.e. total completed trials = 24 minus any that were removed during data cleaning); we examined latency for both correct and incorrect antisaccades.

To further explore the main sequence, we fitted a square root model,^57^ which is a robust model to quantify the main sequence in smaller sample sizes^58^:


y=V√x

where *y* is the peak velocity, *x* is the absolute amplitude and *V* is the coefficient.^58-60^ One thousand bootstrapped estimates of *V* were simulated for each saccade, from which the median *V* was computed.^60^ To determine how the placebo and atomoxetine groups deviated from the controls’ main sequence, for each observed peak velocity value in the patients, we subtracted the modelled velocity value at a matched amplitude, using the curve derived from controls.^60^ This created a Δ peak velocity value, where higher or lower values reflect greater deviation from the normative model.

### Statistical analysis

For all oculomotor measures, we compared people with Parkinson’s disease on atomoxetine versus placebo. Separately, we compared participants with Parkinson’s disease on placebo versus controls. Analyses were conducted in R (version 4.2.1, R Core Team, 2022). We applied linear mixed models constructed using trial-wise data, accounting for repeated measures and pseudoreplication using random effects with a fixed effect of visit order also included. Frequentist models were implemented using the ‘afex’ package^61^ and the ‘emmeans’ package^62^ for *post hoc* comparisons with Sidak adjustments for multiple testing. Bayes factor (BF) analyses were implemented using ‘BayesFactor’^63^ and ‘bayestestR’^64^ packages. We report the BF for the alternative hypothesis over the null hypothesis (i.e. BF_10_), with BF > 1 indicating relative evidence for the alternative hypothesis and BF > 3 indicating positive evidence for the alternative hypothesis and BF < 0.33 indicating positive evidence for the null hypothesis.^65^

## Results

### Demographics and clinical characteristics

Parkinson’s disease and control groups were well matched on demographics, but the patient group scored significantly lower on the ACE-R and its memory subscale (Table 1).

### Resting pupil diameter

**Figure 1:**
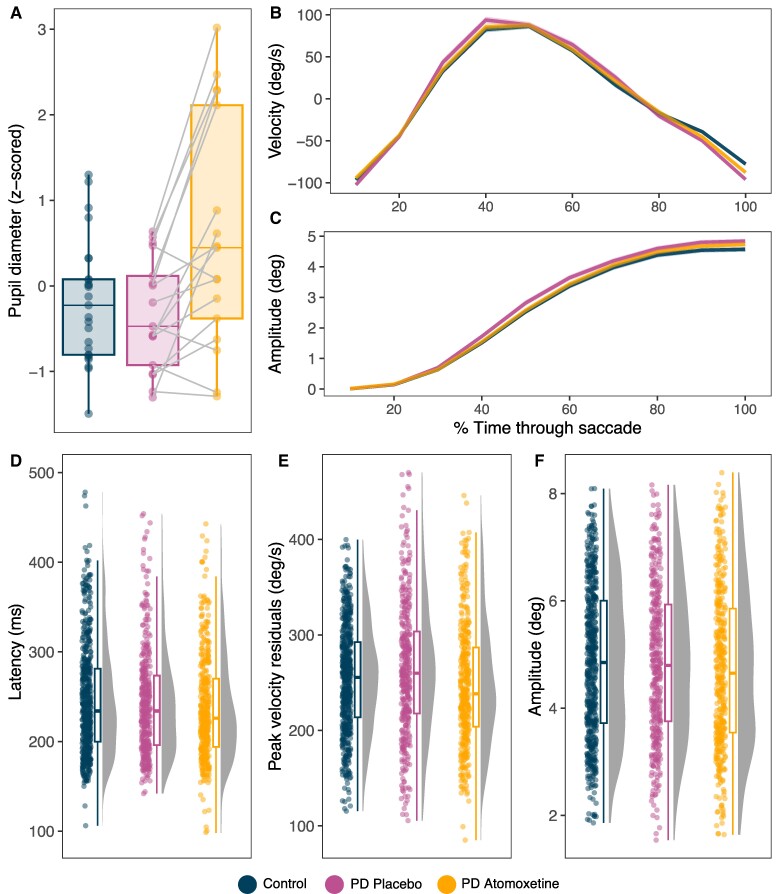
**Resting pupil diameter and prosaccade parameters.** (**A**) Mean *Z*-scored pupil diameter at rest, with lines representing within subject comparisons tested with linear mixed effects models [Parkinson’s disease-atomoxetine versus Parkinson’s disease-placebo: *F*(1, 15) = 16.68, *P* < 0.001, BF = 34.46; Parkinson’s disease-placebo versus control: *F*(1, 40) = 0.43, *P* = 0.517, BF = 0.364]. (**B**) Mean velocity residuals and (**C**) amplitude throughout the time course of prosaccades, where the *x*-axis is % of normalized time with 0 indicating the start of a saccade and 100 indicating the end. Shaded ribbons represent standard error of the mean. (**D**) Latency, (**E**) peak velocity residuals, and (**F**) amplitude of prosaccades for the three groups tested with linear mixed effects model for Parkinson’s disease-atomoxetine versus Parkinson’s disease-placebo [latency: *F*(1, 14.73) = 6.62, *P* = 0.022, BF = 2.29; peak velocity residuals: *F*(1, 16.64) = 0.01, *P* = 0.913, BF = 0.066; amplitude: *F*(1, 16.35) = 1.11, *P* = 0.309, BF = 1.38] and between Parkinson’s disease-placebo versus control [latency: *F*(1, 41.94) = 0.037, *P* = 0.848, BF = 0.211; peak velocity residuals: *F*(1, 42.14) = 0.04, *P* = 0.854, BF = 0.415; amplitude: *F*(1, 41.44) = 0.02, *P* = 0.879, BF = 0.224]. Data points show trial-wise data, representing the primary saccade for every trial per participant. ms, millisecond; deg, degrees; deg/s, degrees travelled per second.

Mean pupil diameter was significantly increased on atomoxetine compared to placebo [*F*(1, 15) = 16.68, *P* < 0.001, BF = 34.46] (Fig. 1A). Mean pupil diameter in the patient-placebo group was not significantly different from controls [*F*(1, 40) = 0.43, *P* = 0.517, BF = 0.364].

### Prosaccade velocity and amplitude over the saccade time course

All groups demonstrated the expected velocity and amplitude trajectories throughout prosaccades, with a symmetric profile of acceleration and deceleration around the peak velocity (Fig. 1B and C). In the Parkinson’s disease group, prosaccade mean velocity residuals and mean amplitude across 10 time bins were not significantly different after atomoxetine versus placebo. There was no interaction between drug condition and time bin and no effect of visit. Comparing the Parkinson’s disease group on placebo with control participants, there was no significant difference in mean velocity and amplitude across the time course, with no interaction between group and time bin (results detailed in Supplementary material).

### Prosaccade latency

There was evidence of faster saccadic responses on atomoxetine (Fig. 1D), with significantly reduced latencies compared to placebo [*F*(1, 14.73) = 6.62, *P* = 0.022, BF = 2.29]. We note that the BF was below conventional threshold for positive evidence (i.e. BF > 3), so this effect should be regarded as ‘anecdotal’. The effect of visit order was not significant [*F*(1, 14.14) = 3.70, *P* = 0.075, BF = 1.16]. Latency did not differ between the Parkinson’s disease group on placebo and control participants [*F*(1, 41.94) = 0.037, *P* = 0.848, BF = 0.211].

### Prosaccade peak velocity and absolute amplitude

For peak velocity residuals (Fig. 1E), there was evidence for no difference on atomoxetine compared to placebo [*F*(1, 16.64) = 0.01, *P* = 0.913, BF = 0.066]. For absolute saccade amplitude (Fig. 1F), there was also evidence for no difference between the conditions [*F*(1, 16.35) = 1.11, *P* = 0.309, BF = 1.38]. With target location included as a covariate to account for the three target distances, there was a main effect of location [*F*(2, 29.04) = 139.770, *P* < 0.001, BF = 1.93 × 10^125^] reflecting larger amplitudes with increasing target distance; there was no interaction between target location and drug condition [*F*(2, 32.91) = 0.14, *P* = 0.874, BF = 0.037]. For both peak velocity and absolute amplitude, there was no significant effect of visit order [peak velocity: *F*(1, 16.65) = 0.11, *P* = 0.749, BF = 0.289; amplitude: *F*(1, 16.02) = 0.18, *P* = 0.674, BF = 0.222].

The Parkinson’s disease group on placebo and controls did not differ in peak velocity residuals [*F*(1, 42.14) = 0.04, *P* = 0.854, BF = 0.415] or absolute amplitude [*F*(1, 41.44) = 0.02, *P* = 0.879, BF = 0.224]. There was the expected effect of target location on amplitude [*F*(2, 75.09) = 501.30, *P* < 0.001, BF = 3.37 × 10^420^] with no interaction between group and target location [*F*(2, 75.01) = 2.04, *P* = 0.138, BF = 0.324].

### Saccade main sequence

**Figure 2:**
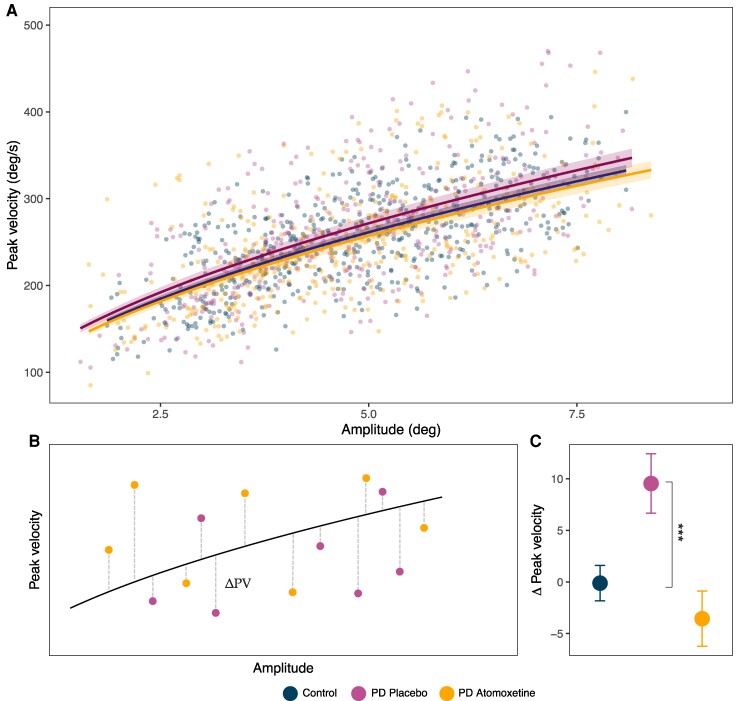
**Prosaccade main sequence.** (**A**) A square root model was fit to estimate the main sequence of prosaccades in each group. Solid lines illustrate the median fit computed over 1000 bootstrapped estimates of the coefficient (*V*), with 95% confidence intervals displayed by the shaded ribbons. (**B**) Schematic of the peak velocity delta (Δ) calculations for each primary prosaccade from the normative main sequence curve (control curve estimated in Fig. 1A). (**C**) Mean Δ peak velocity for the three groups with standard error bars. One-sample *t*-tests for Δ peak velocity were conducted for the Parkinson’s disease-placebo and Parkinson’s disease-atomoxetine groups against zero. Patients on placebo were significantly different from zero [*t* (371) = 3.31, *P* = 0.001, BF = 12.23] while patients on atomoxetine did not differ from zero [*t* (386) = −1.33, *P* = 0.184, BF = 0.14].

To determine the effect of atomoxetine on the main sequence, trial-wise model coefficients (*V*) for each prosaccade were analysed from square root models fitted to each group (Fig. 2A). A significant main effect of the drug was found, suggesting that atomoxetine alters the main sequence in Parkinson’s disease [*F*(1, 15.16) = 20.32, *P* < 0.001, BF = 313.21], with no effect of visit order [*F*(1, 16.64) = 0.004, *P* = 0.951, BF = 0.109]. The main sequence coefficient differed significantly between the Parkinson’s placebo group and controls [*F*(1, 40.88) = 8.17, *P* = 0.007, BF = 1.12]. One-sample *t*-tests tested whether Δ peak velocity values (i.e. the difference between the Parkinson’s disease groups’ observed values and the controls’ modelled main sequence curve) in the atomoxetine and placebo groups differed from zero (Fig. 2B). Δ peak velocity values in the placebo group were significantly different from zero [*t* (371) = 3.31, *P* = 0.001, BF = 12.23], but there was evidence for no difference for the atomoxetine group [*t* (386) = −1.33, *P* = 0.184, BF = 0.14] (Fig. 2C). Together, suggesting that atomoxetine normalized the main sequence to more closely resemble that of controls.

### Antisaccade errors

**Figure 3:**
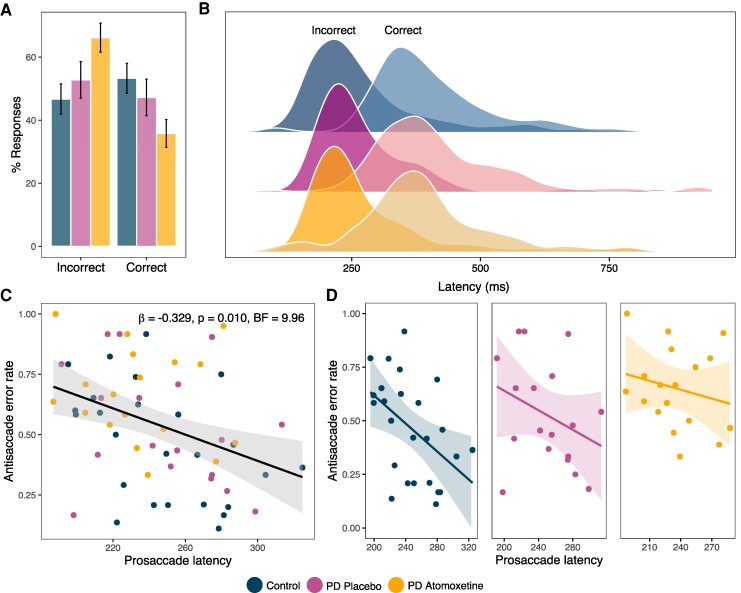
**Antisaccade error and its relationship with prosaccade latency.** (**A**) Antisaccade task % of responses for incorrect and correct trial types. (**B**) Antisaccade task latencies for incorrect (darker distribution on the left) and correct (transparent distribution on the right) trial types. (**C**) Relationship between mean prosaccade latency (millisecond) and antisaccade error rate for all participants coloured by condition, tested with linear mixed effects model [F(1, 53.66) = 7.10, *P* = 0.010, BF = 9.96]. (**D**) For visualization purposes, we also show the relationship between mean prosaccade latency (millisecond) and antisaccade error rate for each group. ms, millisecond.

The proportion of errors in the antisaccade task (Fig. 3A) was increased under atomoxetine compared to placebo [*F*(1, 16) = 7.57, *P* = 0.014, BF = 3.71], with no effect of visit order [*F*(1, 16) = 0.14, *P* = 0.715, BF = 0.419]. Errors did not differ between the Parkinson’s disease group on placebo and control participants [*F*(1, 41) = 0.738, *P* = 0.395, BF = 0.407].

Consistent with antisaccade errors reflecting impulsive responses, when latency was examined with correct versus incorrect trial type included as a covariate, response latencies were significantly faster for incorrect antisaccade trials compared to correct trials (Fig. 3B). This was the case for both atomoxetine versus placebo [*F*(1, 15.91) = 45.64, *P* < 0.001, BF = 3.47 × 10^3^] and placebo versus control [*F*(1, 39.20) = 109.77, *P* < 0.001, BF = 2.99 × 10^9^], with no interaction between trial type and condition [atomoxetine versus placebo: *F*(1, 19.60) = 2.11, *P* = 0.163, BF = 0.408; placebo versus control: *F*(1, 39.20) = 0.44, *P* = 0.511, BF = 0.221].

### Relationship between prosaccade latency and antisaccade error rate

That atomoxetine made prosaccade latency faster, while making antisaccade errors worse is consistent with the established relationship such that prosaccade latency is a reliable predictor of antisaccade error rate.^66,67^ We confirmed this relationship with a general linear mixed model controlling for group as a fixed effect and a random effect of subjects on the intercept [i.e. error rate ∼ latency + group + (1|subject)]. We found a significant relationship between mean prosaccade latency and antisaccade error rate across the population [*F*(1, 53.66) = 7.10, *P* = 0.010, BF = 9.96; Fig. 3C and D], consistent with faster prosaccade latencies associated with higher error rates on the antisaccade task.

### Saccade performance on placebo in relation to disease severity and cognitive impairment

Given the literature showing that saccade deficits in Parkinson’s disease worsen with disease progression and cognitive impairment, we tested whether prosaccade latency, antisaccade error rate or main sequence deviation (i.e. Δ peak velocity values from controls’ main sequence) in the placebo group correlated with global cognition (ACE-R total score), disease severity (MDS-UPDRS-III score) or dopamine medication (dopamine dose equivalent). We conducted Pearson correlations with false discovery rate (FDR) correction applied within both families of comparisons. Both prosaccade latency and main sequence deviation showed a positive correlation with disease severity, consistent with worsening impairment with disease progression (latency: *r* = 0.549, *P*_(adjust)_ = 0.022, BF = 4.81; Δ peak velocity: *r* = 0.692, *P*_(adjust)_ = 0.003, BF = 29.89) (see Supplementary material and Supplementary Fig. 3 for full results).

## Discussion

The noradrenaline reuptake inhibitor atomoxetine altered several aspects of saccade behaviour: prosaccades were faster and the main sequence more closely resembled that of controls. This was accompanied by an increase in antisaccade errors. These findings highlight a role for noradrenergic modulation of oculomotor control in Parkinson’s disease and emphasize a key role for noradrenaline in modulating behavioural decisions, including eye movements. In the context of potential noradrenergic treatments, our findings suggest that oculomotor control may be a sensitive, objective measure to detect drug effects on cognition that are unconfounded by limb-motor deficits.

Atomoxetine had significant effects on the initiation of saccades: faster prosaccade latencies and more antisaccade errors were seen under atomoxetine relative to placebo. Consistent with speed and accuracy trade-off on a shared dimension, we found that higher rates of antisaccade errors were associated with faster prosaccade latencies. Atomoxetine influenced this trade-off in individuals’ exertion of cognitive control, unconfounded by limb-motor deficits in the kinetics of decision execution.

At the heart of saccade control is a negotiation between salient stimuli that trigger automatic responses (e.g. a saccade to the stimulus), versus deliberative control (e.g. an antisaccade). Even a seemingly simple prosaccade towards a target occurs at approximately 200 ms latency in humans—a delay that is much slower than the constraints imposed by anatomy.^68,69^ This has been called the ‘oculomotor procrastination’,^70^ in deciding not only where to look, but whether it is worth looking. For antisaccade tasks, this can be conceptualized as a competition between two processes: supressing a prepotent response towards the target and generating a voluntary saccade in the opposite direction.^1,71^ The balance between issuing a prepotent response versus exerting deliberative control is modulated by noradrenaline. This is most clearly demonstrated by cross-species evidence from the stop-signal task, where atomoxetine can improve the ability to inhibit a prepotent motor response.^72,73^ In a related domain, noradrenaline is implicated in the ability to shift responding from an established attentional set to a new set of responses.^74,75^ In this way, noradrenergic modulation across different timescales influences the ability to flexibly reconfigure behavioural responses based on changing environmental contingencies. In our Parkinson’s disease patients on atomoxetine, an invigorated response to the visual target, as evidenced by increased prosaccade latency, may come at the expense of implementing the cognitive control required to support antisaccades.

Noradrenergic modulation by atomoxetine may act via the locus coeruleus projections to the superior colliculus or to cortical control systems (given the sparsity of noradrenergic terminals in the striatum). At the level of the locus coeruleus, systemic atomoxetine is associated with an increased phasic-to-tonic firing rate in response to sensory stimuli.^76^ This will affect activity in locus coeruleus noradrenergic projections to the intermediate layers of the superior colliculus, which project in turn to the paramedian pontine reticular containing burst neurons responsible for initiating and stopping saccades.^1^ The faster latencies and higher velocities of saccadic responses typically seen with increased arousal^77^ may be mediated in part by elevated noradrenergic activity in the locus coeruleus pathway projecting to the intermediate superior colliculus.^78^ In our patient group, we found increased pupillary diameter on atomoxetine compared to placebo, corroborating the drug’s effect on an index of the ascending arousal system that has been closely linked to locus coeruleus activity.^79^

Atomoxetine may also modulate cognitive control mediated by cortical networks. Descending inputs from the dorsolateral prefrontal cortex, frontal eye fields, supplementary eye fields and parietal eye fields contribute to ‘top-down’ control over saccade performance.^1^ Activity in these regions has been linked with successful inhibition on antisaccade trials^80^ via both direct and indirect projections to the superior colliculus.^81,82^ The dorsolateral prefrontal cortex is especially sensitive to the effects of noradrenergic modulation, with a non-linear ‘inverted-U’ dose-response effect.^83^ This non-linear response is offset by Parkinson’s disease, but to a different degree across different brain regions.^84^ This means that a given oral dose of atomoxetine may optimize decisions mediated by one region while simultaneously driving decision-making processes at another region out of the optimal range. Therefore, even though the same dose improved stop-signal task limb-motor response inhibition in some individuals with Parkinson’s disease,^35,37,85^ it impaired antisaccade accuracy in this study, highlighting that optimal levels of noradrenergic modulation are often task specific. Although both are measures of response inhibition, the stop-signal reaction time task is a measure of action cancellation, while the antisaccade task is akin to action withholding, with distinct neural correlates^86^ that may explain the task-specific differences seen with the same dose of atomoxetine.

Another effect of atomoxetine was on the main sequence, the well-established relationship between a saccade’s amplitude and its velocity. In patients on atomoxetine, the main sequence trajectory was more closely aligned with that of controls and was the only metric that differed significantly between participants with Parkinson’s disease on placebo versus controls. The main sequence in Parkinson’s disease patients on placebo was characterized by a higher peak velocity, as a function of amplitude, compared to healthy controls. Investigations of the main sequence in Parkinson’s disease are limited, with no deviation from controls reported^87-89^ and one study finding a vertical shift in the main sequence curve, similar to what we have shown, in the context of a task.^56^ A compensatory strategy that prioritizes velocity over target accuracy may explain this pattern in our mild cohort.^90,91^ In our mild-moderate patient cohort, a lack of group differences from controls (including in prosaccade latency, velocity, amplitude and antisaccade errors) is consistent with the heterogeneous findings in these saccade parameters in early-stage Parkinson’s disease.^12,92^ In line with this, some features showed a relationship with disease severity: the extent of main sequence deviation from controls and slower latencies both increased with disease severity.

The main sequence describes a highly stereotyped relationship between the amplitude and velocity of saccades, with a linear increase for short saccades that asymptotes as speed saturates for larger saccades.^55,58^ This non-linear property optimizes the speed-accuracy trade-off, reducing the impact of motor noise on faster movements^93^ and thus minimizing the variability in saccade endpoints.^94^ The superior colliculus is an upstream source of these non-linear kinematics.^95-97^ Saccade-related neurons in the intermediate superior colliculus are organized with rostral-caudal topography that corresponds to the amplitude and spatial location of the saccades they generate.^98^ This arrangement is mirrored by a gradient of burst firing properties along the rostral-caudal extent, where distinct firing profiles occur for smaller to larger amplitudes—an organization that could support non-linear dynamics.^95^ Despite its stereotypy, the main sequence has been shown to deviate as a function of arousal,^77,99^ motivation^100^ and in neurological disease.^90,101-104^ Our results raise the novel possibility that the main sequence is sensitive to noradrenergic modulation, which we speculate is mediated by a direct locus coeruleus to superior colliculus pathway.

Atomoxetine may also increase dopamine in the neocortex, due to the paucity of dopamine transporters extracellular dopamine uptake is largely mediated by the noradrenaline transporter.^105,106^ However, mediation of the effects of atomoxetine by cortical dopamine is unlikely: meta-analyses confirm prolonged latency on (versus off) dopamine medication, with no significant effects on velocity or amplitude parameters,^107^ and antisaccade error rate is not affected by dopamine medication.^108^ Furthermore, atomoxetine is unlikely to strongly modulate the primary dopaminergic circuitry implicated in Parkinson’s disease oculomotor deficits, i.e. the basal ganglia pathways that drive substantia nigra inhibition over the superior colliculus, as the noradrenaline transporter is not responsible for significant dopamine reuptake in the striatum.^105^ Together, this past literature argues against a dopaminergic interpretation of our results.

In summary, we demonstrate an effect of atomoxetine on saccadic control, with faster prosaccade latencies and a normalized main sequence trajectory, concurrent with more antisaccade errors which we suggest may reflect disrupted cognitive control from the frontal cortex. These findings highlight a noradrenergic role for oculomotor function relevant to oculomotor decision-making in Parkinson’s disease. Our findings highlight that basic oculomotor function may be a target for noradrenergic modulation in these conditions or provide an additional means of monitoring the effects of noradrenergic interventions. Noradrenergic drugs have potential to address cognitive and behavioural symptoms in Parkinson’s disease and related disorders.

## Supplementary Material

fcae297_Supplementary_Data

## Data Availability

Code and data to reproduce figures and statistical analyses are available through the Open Science Framework (https://osf.io/zcrek/).
